# Non-HDL to HDL cholesterol ratio as a potential biomarker for osteoporosis: A cross-sectional national population study

**DOI:** 10.1097/MD.0000000000048040

**Published:** 2026-03-13

**Authors:** Guo-xu Zhang, Zhi Qian, Xie Li, Bao-qing Yu

**Affiliations:** a Shanghai University of Traditional Chinese Medicine, Pudong New District, Shanghai, China; b Department of Orthopedics, The Seventh People’s Hospital Affiliated to Shanghai University of Traditional Chinese Medicine, Pudong New District, Shanghai, China.

**Keywords:** biomarker, bone mineral density, cholesterol ratio, lipid, NHANES, NHHR, osteoporosis

## Abstract

This cross-sectional study aimed to investigate the association between the non-high-density lipoprotein cholesterol to high-density lipoprotein cholesterol ratio (NHHR) and osteoporosis (OP) among US women. An analysis was conducted using data from 6149 women participating in the National Health and Nutrition Examination Survey (NHANES). Weighted multivariable logistic regression and restricted cubic spline models were applied to evaluate the relationship between NHHR and OP, with adjustments for sociodemographic, clinical, and metabolic covariates. After full adjustment, a significant U-shaped relationship was identified between NHHR and osteoporosis prevalence, with an inflection point at NHHR = 3.371. Below this threshold, each unit increase in NHHR was associated with a 29% reduction in OP risk (odds ratio = 0.71, 95% confidence intervals: 0.60–0.84, *P* < .001). No significant association was observed above this value. Inverse associations were particularly notable in women aged ≥65 years, non-Hispanic Whites, former smokers, and those with higher education or lower physical activity levels. Non-high-density lipoprotein cholesterol to high-density lipoprotein cholesterol ratio demonstrates a nonlinear, threshold-dependent association with osteoporosis risk, supporting its potential role as a novel biomarker for OP in specific subpopulations. Further prospective studies are warranted to clarify causal mechanisms.

## 1. Introduction

Osteoporosis (OP), a systemic skeletal disease characterized by reduced bone mass, degradation of bone microstructure, increased bone fragility, and elevated fracture risk,^[1,2]^ has been defined as a silent disease of the 21st century.^[3]^ It poses a significant public health challenge, particularly in today’s aging society.^[4]^ Elderly women after menopause are particularly susceptible to osteoporosis, as estrogen deficiency results in progressive bone loss and diminished skeletal strength.^[5,6]^ Epidemiological data indicate that approximately 10 million individuals in the United States (US) are diagnosed with OP, with an additional 43 million exhibiting low bone mass,^[7,8]^ and nearly one-third of women aged 50 years and older are at risk of developing the condition.^[4]^ The disease causes an estimated 1.5 million fractures annually in the United States,^[4,9]^ resulting in higher risks of fractures and death, greater medical expenditures, and a substantial deterioration in quality of life.^[10,11]^ Consequently, the focus on OP and its comorbidity with chronic diseases intensifies, as the projected economic burden is substantial: direct annual costs are expected to reach $25.3 billion by 2025,^[12]^ with the cost of osteoporotic fracture treatment expected to approach $50 billion by 2040,^[13,14]^ placing significant strain on both the economy and society. Therefore, early identification and prevention are crucial to minimize the difficulties associated with this major global public health problem.

The connection between indicators of lipid metabolism and OP continues to be a subject of debate in human research.^[15-17]^ While some studies indicate a negative correlation between lipoproteins like low-density lipoprotein cholesterol (LDL-C) and bone mineral density (BMD),^[18,19]^ others report positive correlations^[20]^ or even no significant associations, particularly in postmenopausal women.^[21]^ This inconsistency extends to high-density lipoprotein cholesterol (HDL-C), recognized for its antiatherogenic properties in cardiovascular disease,^[22]^ where findings conflict between positive correlations with BMD^[23]^ and elevated osteoporosis risk, especially among women.^[24]^ This ambiguity highlights the limitation of focusing on single lipid parameters and underscores the need for a comprehensive lipid index. The non-high-density lipoprotein cholesterol to high-density lipoprotein cholesterol ratio (NHHR) emerges as a novel composite indicator that simultaneously incorporates the atherogenic risk factor (non-HDL-C, encompassing all cholesterol present in LDL, VLDL, and remnant lipoproteins) and the protective factor (HDL-C).^[25]^ Consequently, NHHR provides a more integrated assessment of lipid profile status than isolated lipoprotein measurements and has demonstrated superior predictive value for various cardiometabolic conditions, including coronary artery disease, diabetes, and atherosclerosis.^[26-28]^

Nevertheless, in spite of its possibilities, the precise connection between NHHR and OP, particularly its relation to OP in adult women, has not been thoroughly explored. In order to fill these knowledge voids, the National Health and Nutrition Examination Survey (NHANES) dataset was utilized and a comprehensive cross-sectional study was carried out to evaluate the relation between NHHR and OP in US women.

## 2. Methods and materials

### 2.1. Study design and population

This work relied on information derived from NHANES, a nationwide survey that adopts a multistage cross-sectional design to monitor health and nutrition across the US population. Since 1999, the National Center for Health Statistics of the Center for Disease Control and Prevention has carried out this program every 2 years. The National Health and Nutrition Examination Survey uses questionnaires, physical examinations (including mobile medical examinations), laboratory tests, and strict sampling methods to ensure data accuracy. The National Health and Nutrition Examination Survey data are publicly available and exempt from additional ethical approval.

**Figure 1.**
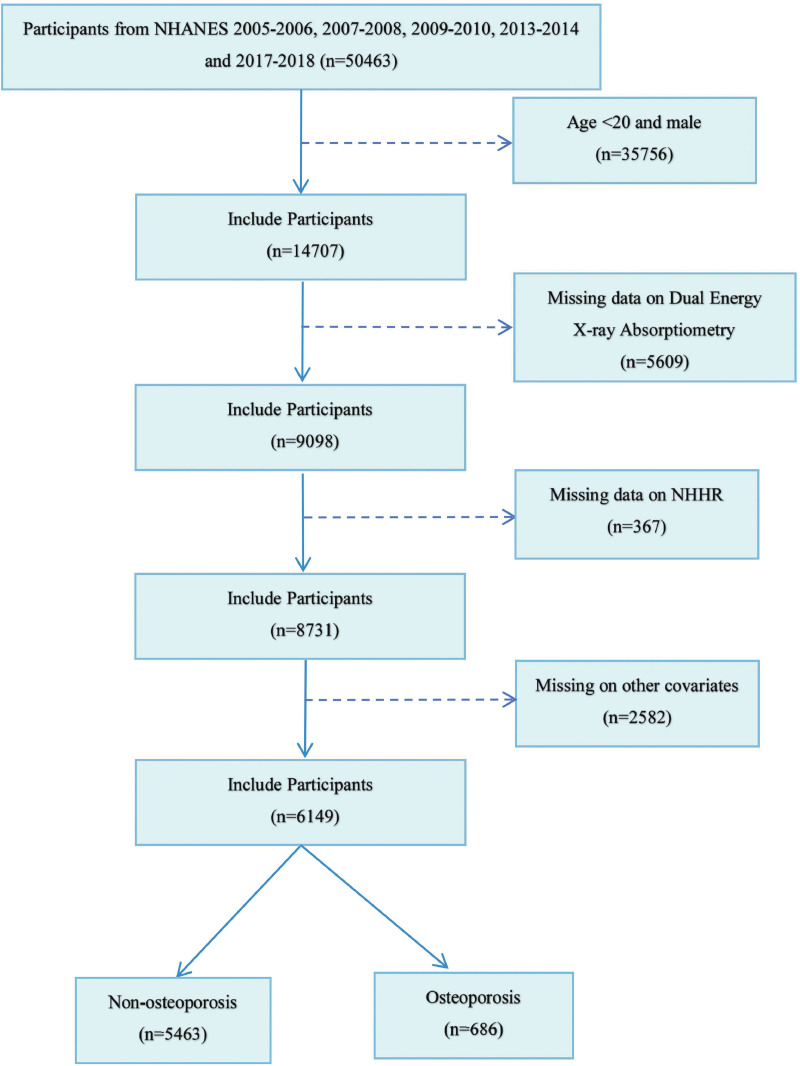
Flow chart of study participant selection from the NHANES 2005 to 2018 cycles. After applying exclusion criteria, the final analytical sample consisted of 6149 women, among whom 686 were diagnosed with osteoporosis. NHANES = National Health and Nutrition Examination Survey, NHHR = non-high-density lipoprotein cholesterol to high-density lipoprotein cholesterol ratio.

This study utilized data from 50,463 individuals in the NHANES 2005 to 2018. Data from the 2011 to 2012 and 2015 to 2016 cycles did not include femoral BMD data and were therefore excluded from the analysis. Among the participants, 14,707 females were over 20 years old, 5609 had missing BMD data, and 2949 had missing NHHR and covariate data. Figure 1 presents the flowchart of the screening process.

### 2.2. Assessment of NHHR

Blood lipid parameters were measured at mobile examination centers. After collection, serum samples were processed, stored, and subsequently sent to the University of Minnesota for analysis. In this study, the non-high-density lipoprotein cholesterol to high-density lipoprotein cholesterol ratio (NHHR) was used as the exposure variable to evaluate lipid profiles and OP risk. Non-high-density lipoprotein cholesterol to high-density lipoprotein cholesterol ratio was calculated from participants’ lipid data obtained from the NHANES database spanning 2005 to 2018. The calculation involved determining non-HDL-C ([total cholesterol {TC} − HDL-C]) and deriving the ratio of non-HDL-C to HDL-C ([NHHR = {TC − HDL-C}/HDL-C]). For analysis, participants were grouped into quartiles based on their NHHR values, where Q1 was used as the reference group, representing the lowest range.

### 2.3. Assessment of osteoporosis

Bone mineral density was assessed in all participants using dual-energy x-ray absorptiometry at the proximal femur. All examinations were performed by trained radiologic technologists with the Hologic QDR-4500A fan-beam densitometer (Hologic, Bedford). Detailed descriptions of the dual-energy x-ray absorptiometry methodology can be accessed on the NHANES official platform. According to the World Health Organization criteria, the BMD *T*-score is defined as the deviation of the measured BMD from the average BMD of a young healthy reference group.^[29]^ The reference standard used the mean BMD of non-Hispanic White women aged 20 to 29 years.^[30]^ Osteoporosis was defined as a femoral neck BMD that was 2.5 standard deviations or more below the reference mean.

### 2.4. Assessment of covariates

Physical activity levels were assessed using weekly metabolic equivalent task (MET)-minute scores, which were calculated from the Global Physical Activity Questionnaire. Following the NHANES guidelines, weekly MET-minutes were derived as follows: [8.0 METs × (weekly minutes of vigorous work-related activity + weekly minutes of vigorous leisure-time physical activity)] + [4.0 METs × (weekly minutes of moderate work-related activity + weekly minutes of moderate leisure-time physical activity + weekly minutes of walking or bicycling for transportation)]. Participants were classified into 3 activity levels based on criteria from previous studies: inactive/insufficiently active: <600 MET-min/wk; moderately active: 600–3000 MET-min/wk; highly active: >3000 MET-min/wk.^[31]^ Smoking status was determined from self-reported responses to 2 questions: lifetime consumption of ≥100 cigarettes and current smoking status. Participants who reported smoking ≥100 cigarettes but were not current smokers were categorized as former smokers. The analysis adjusted for the following covariates: age, race, poverty income ratio (PIR), education level, diabetes status, body mass index (BMI), chronic kidney disease (CKD), alcohol consumption history, history of prednisone/cortisone use, total protein, alkaline phosphatase, aspartate aminotransferase (AST), alanine aminotransferase (ALT), creatinine, blood urea nitrogen (BUN), calcium, phosphorus, and 25-hydroxyvitamin D levels in serum. Comprehensive details on the measurement procedures and data collection for each variable can be accessed through the NHANES official website (www.cdc.gov/nchs/nhanes).

### 2.5. Statistical analyses

Following Centers for CDC guidelines, appropriate sampling weights were applied throughout all analyses. Continuous data are summarized as weighted median and interquartile range, whereas categorical data are represented by weighted percentages or frequencies. Group comparisons for continuous measures utilized the Kruskal–Wallis test; for categorical measures, the χ^2^ test was applied. Three weighted multivariate logistic regression models were established to estimate the relationship between NHHR and OP. Weighted restricted cubic splines (RCS) facilitated the examination of potential linear and nonlinear associations linking NHHR to OP. Subgroup analyses, complemented by interaction testing, assessed the robustness of the NHHR-OP association across diverse population strata. R software (version 4.4.2; The R Foundation for Statistical Computing, Vienna, Austria) was employed for all statistical procedures, with statistical significance determined by a 2-tailed *P*-value below .05. Participants with missing data on BMD, NHHR, or any of the included covariates were excluded from the final analysis. This complete-case approach was adopted as the primary analytic strategy.^[32]^

## 3. Results

### 3.1. Characteristics of the study population

Table 1 presents the characteristics of the 6149 participants, including 686 diagnosed with OP. Compared with the non-OP group, the OP group was older and had a higher prevalence of self-reported diabetes, a history of CKD, and previous glucocorticoid (prednisone/cortisone) use. Education level, serum calcium, serum phosphorus, ALT, and smoking status were similar between the OP and non-OP groups. However, significant differences were observed in race/ethnicity, PIR, alcohol consumption, BMI, total protein, creatinine, BUN, alkaline phosphatase, AST, serum 25-hydroxyvitamin D levels, and physical activity levels. Additionally, the NHHR was significantly lower in the OP group compared to the non-OP group.

**Table 1:** Participant characteristics by osteoporosis status.

Characteristic	Overall, N = 6149 (100%)*	Non-osteoporosis, N = 5463 (89%)*	Osteoporosis, N = 686 (11%)*	*P*-value†
Age				<.001
<65	4423 (76%)	4188 (80%)	235 (40%)	
≥65	1726 (24%)	1275 (20%)	451 (60%)	
Race/ethnicity				<.001
Mexican American	942 (7%)	876 (7%)	66 (4%)	
Non-Hispanic White	2971 (73%)	2557 (72%)	414 (80%)	
Non-Hispanic Black	1081 (10%)	1036 (11%)	45 (3%)	
Other Hispanic	636 (5%)	576 (5%)	60 (4%)	
Other race	519 (6%)	418 (6%)	101 (10%)	
Education				.034
Lower than high school	1451 (15%)	1274 (15%)	177 (17%)	
High school graduate/GED	1403 (23%)	1225 (23%)	178 (27%)	
Above high school graduate	3295 (62%)	2964 (62%)	331 (56%)	
PIR				.001
<1.3	1909 (20%)	1696 (20%)	213 (21%)	
1.3–3.5	2352 (36%)	2053 (35%)	299 (45%)	
>3.5	1888 (44%)	1714 (45%)	174 (35%)	
Diabetes				.045
Yes	747 (9%)	641 (9%)	106 (12%)	
No	5402 (91%)	4822 (91%)	580 (88%)	
CKD				.002
Yes	175 (2%)	141 (2%)	34 (5%)	
No	5974 (98%)	5322 (98%)	652 (95%)	
Drinking history				.009
Yes	4757 (82%)	4274 (83%)	483 (78%)	
No	1392 (18%)	1189 (17%)	203 (22%)	
Smoking status				.7
Never smoker	3806 (61%)	3378 (61%)	428 (59%)	
Former smoker	1247 (22%)	1096 (22%)	151 (21%)	
Current smoker	1096 (18%)	989 (18%)	107 (19%)	
History of prednisone/cortisone				.015
Yes	386 (7%)	329 (6%)	57 (10%)	
No	5763 (93%)	5134 (94%)	629 (90%)	
BMI (kg/m^2^)	27.20 (23.43–31.96)	27.56 (23.70–32.41)	24.80 (21.64–28.10)	<.001
Creatinine (mg/dL)	0.76 (0.67–0.86)	0.75 (0.67–0.86)	0.78 (0.69–0.92)	<.001
BUN (mg/dL)	12.00 (10.00–16.00)	12.00 (10.00–15.00)	15.00 (11.00–18.00)	<.001
ALT (U/L)	18.00 (15.00–23.00)	18.00 (15.00–23.00)	18.00 (14.00–23.00)	.2
AST (U/L)	22.00 (19.00–26.00)	21.00 (18.00–25.00)	22.00 (19.00–27.00)	.002
Total protein (g/dL)	7.00 (6.70–7.30)	7.00 (6.80–7.30)	6.90 (6.60–7.20)	<.001
Alkaline phosphatase (U/L)	65.00 (53.00–81.00)	65.00 (52.00–80.00)	73.00 (59.00–87.00)	<.001
Serum calcium (mg/dL)	9.40 (9.20–9.60)	9.40 (9.20–9.60)	9.40 (9.20–9.60)	.5
Serum phosphorus (mg/dL)	3.80 (3.50–4.20)	3.80 (3.50–4.20)	3.90 (3.50–4.20)	.079
Serum 25-hydroxyvitamin D (nmol/L)	73.00 (53.50–93.20)	72.30 (53.00–92.70)	79.50 (56.20–97.00)	<.001
Physical activity level				<.001
MET min < 600	2916 (43%)	2485 (42%)	431 (57%)	
MET min 600–3000	1951 (34%)	1775 (35%)	176 (29%)	
MET min > 3000	1282 (22%)	1203 (23%)	79 (14%)	
NHHR	2.33 (1.77–3.20)	2.36 (1.77–3.22)	2.16 (1.69–2.96)	.008

ALT = alanine aminotransferase, AST = aspartate aminotransferase, BMI = body mass index, BUN = blood urea nitrogen, CKD = chronic kidney disease, GED = General Educational Development, MET = metabolic equivalent task, NHHR = non-high-density lipoprotein cholesterol to high-density lipoprotein cholesterol ratio, PIR = poverty income ratio.

* n (unweighted) (%); median (Q1–Q3).

† Pearson χ^2^: Rao & Scott adjustment; design-based Kruskal–Wallis test.

### 3.2. Relationship between the NHHR and OP

Table 2 summarizes the logistic regression outcomes assessing the link between NHHR and osteoporosis, where NHHR was evaluated in 2 ways: as a continuous parameter and by stratification into quartiles. In the unadjusted model, NHHR as a continuous variable did not show a significant association with OP (odds ratio [OR] = 0.92, 95% confidence intervals [CI]: 0.82–1.03, *P* = .13). However, after adjusting for covariates including age, race, education level, PIR, diabetes, alcohol consumption, smoking, CKD group, physical activity level, and history of prednisone or cortisone use (model 1), each 1-unit increase in NHHR was significantly associated with a 13% reduction in the prevalence of OP (OR = 0.87, 95% CI: 0.77–0.98, *P* = .024). Even after controlling for variables related to metabolism, the inverse association continued to exist, as indicated in model 2 (OR = 0.87, 95% CI: 0.77–0.99, *P* = .03). When NHHR was divided into quartiles, with Q1 as the control group, significant associations emerged in the higher quartiles after adjustment. In the unadjusted model, the prevalence of OP was significantly lower in Q3 compared with Q1 (OR = 0.72, 95% CI: 0.55–0.94, *P* = .016). After adjustment in model 1, both Q3 (OR = 0.65, 95% CI: 0.48–0.87, *P* = .005) and Q4 (OR = 0.63, 95% CI: 0.44–0.89, *P* = .011) demonstrated a significantly lower prevalence of OP compared to Q1. After comprehensive adjustment of covariates in model 2, the observed inverse associations persisted and remained statistically significant.

**Table 2:** Associations between the NHHR and osteoporosis.

	Crude			Model 1			Model 2		
	OR	95% CI	*P*	OR	95% CI	*P*	OR	95% CI	*P*
NHHR	0.92	0.82–1.03	.13	0.87	0.77–0.98	.024	0.87	0.77–0.99	.03
NHHR (quartile)									
Q1	Reference			Reference			Reference		
Q2	1.10	0.82–1.48	.50	0.99	0.70–1.40	>.9	1.02	0.72–1.46	>.9
Q3	0.72	0.55–0.94	.016	0.65	0.48–0.87	.005	0.66	0.50–0.88	.006
Q4	0.75	0.53–1.05	.093	0.63	0.44–0.89	.011	0.65	0.45–0.95	.026
*P* for trend	.011			<.001			.003		

NHHR: Q1 (<1.767), Q2 (−1.767 to 2.329), Q3 (2.329–3.196), and Q4 (≥3.196). Crude: no parameter was adjusted. Model 1: Adjusted for age, race, education, PIR, diabetes, drinking history, smoking status, CKD group, physical activity level, and history of prednisone or cortisone. Model 2: Additionally adjusted for metabolism related covariates, total protein, alkaline phosphatase, serum calcium, serum phosphorus, ALT, AST, creatinine, BUN, serum 25-hydroxyvitamin D.

ALT = alanine aminotransferase, AST = aspartate aminotransferase, BUN = blood urea nitrogen, CI = confidence interval, CKD = chronic kidney disease, NHHR = non-high-density lipoprotein cholesterol to high-density lipoprotein cholesterol ratio, OR = odds ratio, PIR = poverty income ratio.

A significant inverse trend in OP prevalence was observed across all models as NHHR quartiles increased (*P* for trend < 0.05). Consistent and significant *P* values for trend in model 2, even after comprehensive adjustment for potential confounders, including metabolic factors, underscore the robust dose–response relationship between higher NHHR quartiles and decreased prevalence of OP.

### 3.3. The nonlinear relationship between the NHHR and OP

**Figure 2.**
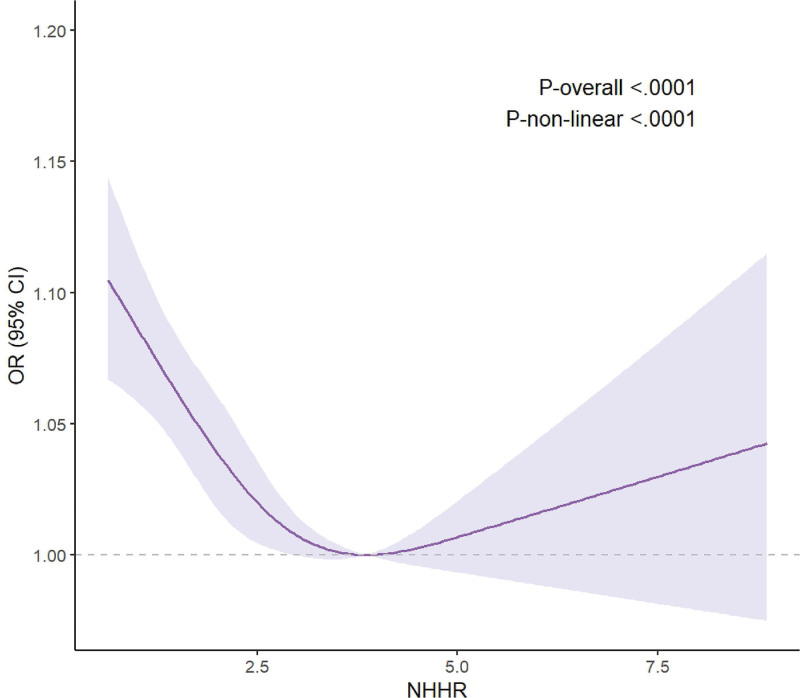
Nonlinear association between NHHR and osteoporosis risk among US women (n = 6149), modeled using weighted restricted cubic splines (RCS) with 3 knots. The solid curve represents the adjusted odds ratio (OR); the shaded band denotes the 95% confidence interval. The model (model 2) was adjusted for age, race, education, PIR, diabetes, alcohol consumption, smoking, CKD, physical activity, glucocorticoid use, total protein, alkaline phosphatase, serum calcium, serum phosphorus, ALT, AST, creatinine, BUN, and serum 25-hydroxyvitamin D. ALT = alanine aminotransferase, AST = aspartate aminotransferase, BUN = blood urea nitrogen, CI = confidence interval, CKD = chronic kidney disease, NHHR = non-high-density lipoprotein cholesterol to high-density lipoprotein cholesterol ratio, OR = odds ratio, PIR = poverty income ratio, RCS = restricted cubic splines, US = United States.

In adjusted model 2, we applied weighted RCS analysis to assess the potential dose–response association between NHHR and OP. The results revealed a statistically significant U-shaped curve (Fig. 2). The turning point of this curve was located at an NHHR level of 3.371, supported by a log-likelihood ratio test (*P* = .001). When NHHR values were below 3.371, an inverse relationship was evident, with each 1-unit rise in NHHR corresponding to a 29% lower risk of osteoporosis (OR = 0.71, 95% CI: 0.602–0.837, *P* < .001). Conversely, when the ratio exceeded 3.371, the estimated effect size was OR = 1.109 (95% CI: 0.94–1.309, *P *= .221), indicating no statistically meaningful association (Table 3).

**Table 3:** Threshold effect analysis of the NHHR and OP.

Osteoporosis	Adjusted OR (95% CI), *P* value
NHHR	
Inflection point	3.371
NHHR < inflection point	0.71 (0.602–0.837), <.001
NHHR > inflection point	1.109 (0.94–1.309), .221
Log-likelihood ratio	.001

Model 2: Adjusted for age, race, education, PIR, diabetes, drinking history, smoking status, CKD group, physical activity level, history of prednisone or cortisone, total protein, alkaline phosphatase, serum calcium, serum phosphorus, ALT, AST, creatinine, BUN, and serum 25-hydroxyvitamin D.

ALT = alanine aminotransferase, AST = aspartate aminotransferase, BUN = blood urea nitrogen, CI = confidence interval, CKD = chronic kidney disease, NHHR = non-high-density lipoprotein cholesterol to high-density lipoprotein cholesterol ratio, OP = osteoporosis, OR = odds ratio, PIR = poverty income ratio.

### 3.4. Subgroup analysis and interaction test

**Figure 3.**
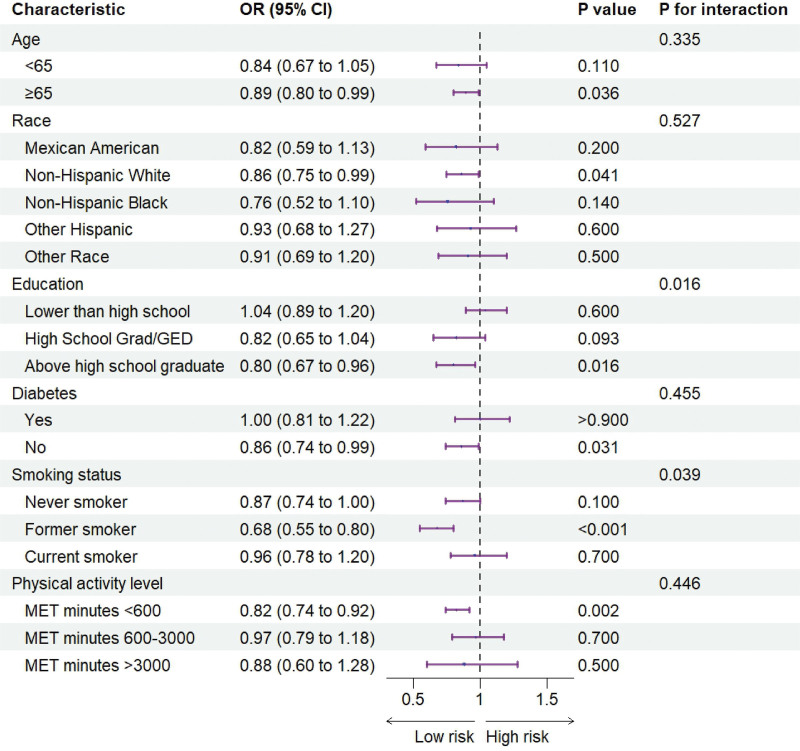
Subgroup analysis of the association between NHHR (per 1-unit increase) and osteoporosis risk among 6149 US women. Odds ratios and 95% confidence intervals are derived from weighted multivariable logistic regression models stratified by the specified subgroups. All models included the core covariates from model 2 (see Fig. 2 legend). The *P*-value for interaction was calculated to test effect modification across subgroups. CI = confidence interval, MET = metabolic equivalent task, NHHR = non-high-density lipoprotein cholesterol to high-density lipoprotein cholesterol ratio, US = United States.

A subgroup analysis was performed to assess the association between the NHHR and OP risk across different demographic and clinical characteristics (Fig. 3). A negative association between the NHHR and OP was recorded in participants who were over 65 years old (OR = 0.89, 95% CI: 0.80–0.99), non-Hispanic White participants (OR = 0.86, 95% CI: 0.75–0.99), with former smoker (OR = 0.68, 95% CI: 0.55–0.80), with a higher education level (OR = 0.80, 95% CI: 0.67–0.96), with no diabetes (OR = 0.86, 95% CI: 0.74–0.99) and with lower physical activity levels (MET minutes < 600; OR = 0.82, 95% CI: 0.74–0.92) was more obvious. Both educational attainment and smoking status showed significant interactions.

## 4. Discussion

To the best of our knowledge, this research is the first to show a remarkable opposite relation between NHHR and the incidence of OP in adult women in the United States by using data from the nationally representative NHANES. This study, after making full adjustments for confounding factors, shows that there is a U-shaped link between NHHR and OP in adult female participants in the United States, which offers new perspectives on the intricate interactions between atherosclerotic lipid profiles and skeletal health.

In the unadjusted model, the NHHR was not significantly associated with OP (OR = 0.92, *P* = .13). This lack of association is possibly attributable to substantial confounding by age and comorbidities. Notably, the OP group was significantly older and exhibited higher prevalence of diabetes, CKD, and corticosteroid use – factors strongly linked to dyslipidaemia and accelerated bone loss. Crucially, upon adjustment for these and other demographic, socioeconomic, and clinical covariates in model 1, a significant inverse relationship emerged: each 1-unit increment in NHHR was associated with a 13% reduction in osteoporosis prevalence (OR = 0.87, 95% CI: 0.77–0.98, *P* = .024). This association persisted robustly after further adjustment for metabolic-related factors in model 2 (OR = 0.87, 95% CI: 0.77–0.99, *P* = .03). Analysis by NHHR quartiles further confirmed this inverse correlation, demonstrating a significant trend of decreasing osteoporosis risk across increasing quartiles (*P*-trend < .05 for all models). In the fully adjusted model 2, participants in Q4 had a 37% lower risk of osteoporosis compared to those in Q1 (OR = 0.63, 95% CI: 0.44–0.89, *P* = .026). This consistent dose–dependent pattern strengthens the biological plausibility of NHHR as a potential marker for bone health status.

An important new finding is the identification of a significant U-shaped nonlinear relationship between NHHR and OP risk. The inflection point is at NHHR = 3.371. Below this threshold, NHHR has a significant protective effect against OP (OR = 0.71, 95% CI: 0.602–0.837, *P* < .001). However, when NHHR exceeds 3.371, no significant association is observed (OR = 1.109, 95% CI: 0.94–1.309, *P* = .221). This suggests that while a moderate increase in NHHR (reflecting a higher level of non-HDL-C relative to HDL-C) may be associated with a reduced risk of OP, excessively high levels lose this protective effect. This nonlinear relationship integrates previous conflicting research evidence on the interaction between individual lipid components (HDL-C and LDL-C) and BMD. For instance, a study investigating Italian clinic and community populations found a positive correlation between HDL-C and BMD, and a negative correlation between LDL-C and BMD.^[33]^ However, a cross-sectional study of 373 postmenopausal women in Russia revealed that the association between HDL-C and bone mass was mediated by other factors, and multivariate regression analysis failed to confirm the relationship between BMD and HDL-C.^[17]^ In another cross-sectional survey of 1116 Chinese women, it was discovered that the relationship between LDL-C, HDL-C, and lumbar spine BMD in postmenopausal women was nonlinear.^[19]^ When LDL-C was <3.52 mmol/L and HDL-C was <2.37 mmol/L, both were negatively correlated with lumbar spine BMD.^[19]^ These differences may stem from differences in the study population, bone density assessment methods, and the complex bidirectional effects of lipids on bone metabolism. The threshold effect of NHHR may reflect its dual role in balancing atherosclerotic (non-HDL-C) and anti-atherosclerotic (HDL-C) lipids, which collectively influence skeletal homeostasis through multiple pathways. Mechanistically, HDL-C promotes osteoblast differentiation and mineralization through the Wnt/β-catenin and AMPK signaling pathways, and inhibits osteoclast generation by suppressing activation.^[34-36]^ In contrast, LDL-C, particularly its oxidized form (OxLDL), increases reactive oxygen species (ROS) levels, activates osteoclast-inducing pathways (such as the RANKL/OPG axis and the RhoA/ROCK pathway), and disrupts osteoblast function by inhibiting the expression of bone morphogenetic protein-2 (BMP-2) and Runx2.^[37-39]^ At lower NHHR levels (driven by higher HDL-C), the protective effect predominates; above the threshold, accumulated non-HDL-C components (such as LDL-C) may offset these benefits, exacerbating bone resorption and inflammation.

Our findings differ from the analysis by Wang et al^[21]^ based on NHANES data. That study reported an L-shaped negative correlation between NHHR and BMD in US adults, whereas our study revealed a U-shaped relationship between NHHR and osteoporosis risk in a female-specific cohort (inflection point NHHR = 3.371). This discrepancy may stem from several factors: First, our study focused exclusively on adult women, where osteoporosis exhibits higher prevalence and distinct pathophysiological mechanisms. Gender-specific analyses are crucial for understanding lipid-bone interactions. Second, Wang et al^[21]^ examined only the relationship between NHHR and lumbar spine BMD, without converting BMD to diagnostic criteria for osteoporosis. In contrast, we defined osteoporosis based on WHO criteria using femoral neck *T*-scores, which carry greater clinical diagnostic significance. Finally, Wang et al^[21]^ included fewer covariates, all of which were categorical variables. In contrast, our logistic regression model adjusted for a more comprehensive set of covariates, including bone metabolism-related markers (such as alkaline phosphatase, calcium, phosphorus, and vitamin D) and physical activity level indicators, thereby helping to reduce potential confounding factors. The U-shaped association identified in this study provides a more nuanced perspective for understanding the complex relationship between lipids and skeletal health. Nonetheless, several caveats merit attention. First, being cross-sectional, our study cannot establish causality and is subject to both reverse causation and temporal ambiguity. Lipid levels may be influenced by existing bone health status, related medications, or activity limitations, while the single-time-point design prevents ascertaining whether NHHR changes precede or follow bone loss. Therefore, longitudinal data are needed to clarify the temporal sequence and causal direction of the association. Second, although rigorous adjustments were made for a wide range of potential confounders, residual confounding cannot be fully excluded; factors such as dietary fat intake, menopausal status, or hormone replacement therapy may still exert unmeasured influence. Finally, as the analysis was confined to women in the United States, caution is warranted in extrapolating these results to men, to populations with different ethnic backgrounds, or to cohorts from non-US settings. Future prospective studies in diverse populations are required to validate and extend these observations.

## 5. Conclusion

In conclusion, NHHR demonstrates a nonlinear, threshold-dependent association with osteoporosis risk, underscoring its role as a biomarker for lipid-bone crosstalk. Future prospective cohort studies and randomized controlled trials targeting lipid profiles are crucial to confirm causality, elucidate underlying mechanisms, and determine if modulating NHHR within a specific range could be a viable strategy for osteoporosis prevention or management.

## Acknowledgments

The NHANES study protocol received approval from the Institutional Review Board of the National Center for Health Statistics (NCHS). All enrolled participants provided written informed consent during the survey, encompassing consent for both study participation and potential publication of non-identifiable data. As the present investigation constitutes a secondary analysis of the de-identified, publicly available NHANES dataset, no additional ethical review by the authors’ institutional ethics committee was required.

## Author contributions

**Data curation:** Guo-xu Zhang, Zhi Qian.

**Formal analysis:** Guo-xu Zhang, Xie Li.

**Funding acquisition:** Zhi Qian, Bao-qing Yu.

**Methodology:** Zhi Qian, Bao-qing Yu.

**Visualization:** Zhi Qian, Xie Li.

**Writing – original draft:** Guo-xu Zhang.

**Writing – review & editing:** Bao-qing Yu.
